# The Role of Gut Microbial β-Glucuronidase in Estrogen Reactivation and Breast Cancer

**DOI:** 10.3389/fcell.2021.631552

**Published:** 2021-08-12

**Authors:** Yue Sui, Jianming Wu, Jianping Chen

**Affiliations:** ^1^School of Chinese Medicine, The University of Hong Kong, Hong Kong, China; ^2^Sichuan Key Medical Laboratory of New Drug Discovery and Druggability Evaluation, Luzhou Key Laboratory of Activity Screening and Druggability Evaluation for Chinese Materia Medica, School of Pharmacy, Southwest Medical University, Luzhou, China; ^3^Shenzhen Institute of Research and Innovation, The University of Hong Kong, Shenzhen, China

**Keywords:** gut microbial β-glucuronidase, estrogen reactivation, breast cancer, host-microbe interaction, gut microbiota, estrogen glucuronide

## Abstract

Over the past decade, the gut microbiota has received considerable attention for its interactions with the host. Microbial β-glucuronidase generated by this community has hence aroused concern for its biotransformation activity to a wide range of exogenous (foreign) and endogenous compounds. Lately, the role of gut microbial β-glucuronidase in the pathogenesis of breast cancer has been proposed for its estrogen reactivation activity. This is plausible considering that estrogen glucuronides are the primary products of estrogens’ hepatic phase II metabolism and are subject to β-glucuronidase-catalyzed hydrolysis in the gut *via* bile excretion. However, research in this field is still at its very preliminary stage. This review outlines the biology of microbial β-glucuronidase in the gastrointestinal tract and elaborates on the clues to the existence of microbial β-glucuronidase–estrogen metabolism–breast cancer axis. The research gaps in this field will be discussed and possible strategies to address these challenges are suggested.

## Introduction

With the microbiota–host interactions being rapidly explored, the microbial metabolic products are considered as the significant mediators within this interplay that are gaining attention, among which, gut microbial β-glucuronidase (gmGUS) is one of the most studied. β-glucuronidase (GUS) has been known to be present in mammalian feces since the early 1970s (Creekmore et al., 2019), and the crystal structure of gmGUS was first reported in 2010 (Wallace et al., 2010). A high frequency of GUS genes has been identified in the human gut-associated microbial genomes (Wallace et al., 2010; Gloux et al., 2011; Kwa et al., 2016; Pollet et al., 2017). For most mammals, such as the human and mouse, the conservation of gmGUS in the gastrointestinal (GI) tract covers the major GI bacterial phyla: Bacteroidetes, Firmicutes, Verrucomicrobia, and Proteobacteria. Among them, Bacteroidetes and Firmicutes, which are dominant in the GI tract, are also responsible for the primary source of gmGUS (Table 1; McIntosh et al., 2012; Pollet et al., 2017; Creekmore et al., 2019; Walsh et al., 2020).

**TABLE 1:** Bacteria from *Bacteroidetes* and *Firmicutes* phylum in the human gastrointestinal tract that encode GUS.

**Phylum**	**Genus**	**Species that encode GUS or GUS candidate in The Human Microbiome Project (*S30*) database Wallace et al., 2010**	**Species that encode GUS in The Human Microbiome Project database Kwa et al., 2016**	Species that encode GUS in the non-redundant Clustered Gene Indices of The Human Microbiome Project database* Pollet et al., 2017	**Species that have been shown to exhibit GUS activity in culture Pellock and Redinbo, 2017**	**Species that encode GUS with reactivating estrogen activities were confirmed by *in vitro* assay Ervin et al., 2019b**
Bacteroidetes	*Alistipes*	*A. putredinis*				
			*A. shahii*	*A. shahii*		
				*A. senegalensis*		
				*A. timonensis*		
	*Bacteroides*	*B. caccae*		*B. caccae*		
		*B. capillosus*			*B. capillosus*	
				*B. clarus*		
		*B. cellulosilyticus*		*B. cellulosilyticus CAG:158*		
		*B. coprocola*		*B. coprocola CAG:162*		
		*B. coprophilus*		*B. coprophilus*		
		*B. dorei*		*B. dorei*		
		*B. eggerthii*		*B. eggerthii*		
		*B. fragilis*		*B. fragilis*	*B. fragilis*	*B. fragilis*
		*B. finegoldii*	*B. finegoldii*	*B. finegoldii*		
		*B. intestinalis*	*B. intestinalis*	*B. intestinalis CAG:564*		
				*B. intestinihominis*		
				*B. massiliensis*		
				*B. msp*		
				*B. nordii*		
		*B. ovatus*	*B. ovatus*	*B. ovatus*	*B. ovatus*	
		*B. plebeius*				
		*Bacteroides* sp. 1_1_6; 2_1_7; 2_2_4; 3_2_5; 4_3_47FAA; 9_1_42FAA; D1; D2; D4	*Bacteroides* sp.	*Bacteroides* sp. CAG:709; CAG:754; HPS0048		
				*B. salyersiae*		
		*B. stercoris*				
				*B. stercorirosoris*		
		*B. thetaiotaomicron*		*B. thetaiotaomicron*	*B. thetaiotaomicron*	
		*B. uniformis*	*B. uniformis*	*B. uniformis*	*B. uniformis*	*B. uniformis*
		*B. vulgatus*		*B. vulgatus*	*B. vulgatus*	
			*B. xylanisolvens*			
				*Candidatus bacteroides timonensis*		
	*Coprobacter*			*C. secundus*		
	*Gabonia*			*G. massiliensis*		
	*Odoribacter*			*O. laneus*		
	*Parabacteroides*			*P. goldsteinii*		
		*P. johnsonii*		*P. johnsonii*	*P. johnsonii*	
		*P. merdae*		*P. merdae*	*P. merdae*	
	*Prevotella*	*P. copri*		*P. copri*		
				*Prevotella* sp. CAG:732; CAG:386		
	*Paraprevotella*			*P. clara*		
	*Propionibacterium*		*Propionibacterium* sp.			
	*Tannerella*		*Tannerella* sp.	*Tannerella* sp. CAG:51		
Firmicutes	*Anaerotruncus*			*Anaerotruncus* sp. CAG:528		
	*Blautia*	*B. hansenii*				
		*B. hydrogenotrophicus*				
	*Butyrivibrio*			*Butyrivibrio* sp. CAG:318	*B. formatexigens*	
	*Clostridiales*	*Clostridiales* sp. 1_7_47FAA		*Clostridiales bacterium KLE1615*		
	*Clostridium*	*C. asparagiforme*	*C. asparagiforme*			
					*C. bartlettii*	
					*C. bifermentans*	
		*C. bolteae*				
					*C. butyricum*	
			*C. celatum*			
			*C. citroniae*			
					*C. clostridioforme*	
		*C. hathewayi*	*C. hathewayi*			
		*C. hylemonae*				
		*C. leptum*				
		*C. nexile*				
					*C. paraputrificum*	
			*C. perfringens*		*C. perfringens*	*C. perfringens*
		*C. ramosum*				
		*C. scindens*				
		*Clostridium* sp. 7_2_43FAA; L2-50; SS2/1	*Clostridium* sp.	*Clostridium* sp. CAG:253; CAG:217; CAG:307; CAG:62; CAG:75; CAG:91		*Clostridium* sp. *Marseille-P299*
		*C. spiroforme*				
	*Catenibacterium*	*C. mitsuokai*				
	*Coprococcus*	*C. comes*				
		*C. eutactus*				
	*Dorea*	*D. formicigenerans*				
		*D. longicatena*		*D. longicatena*		
	*Enterococcus*				*E. faecalis*	
					*E. faecium*	
	*Eubacterium*	*E. eligens*		*E. eligens* CAG:72		*E. eligens*
		*E. hallii*				
					*Eubacterium* L-8	
		*E. rectale*				
		*E. ventriosum*				
				*Eubacterium* sp. CAG: 38; CAG: 76; CAG:115; CAG:180; CAG:251		
	*Faecalibacterium*	*F. prausnitzii*	*F. prausnitzii*	*F. prausnitzii*	*F. prausnitzii*	*F. prausnitzii*
				*Faecalibacterium* sp. CAG:74; CAG:82		
	*Fusicatenibacter*			*Fusicatenibacter*		
				*Fusicatenibacter saccharivorans*		
	*Fusobacterium*	*F. mortiferum*				
	*Holdemania*	*H. filiformis*				
	*Lactobacillus*				*L. acidophilus*	
		*L. brevis*	*L. brevis*			
					*L. gasseri*	
		*L. rhamnosus*	*L. rhamnosus*			*L. rhamnosus*
	*Marvinbryantia*		*M. formatexigens*			
	*Mitsuokella*	*M. multacida*				
	*Roseburia*			*R. hominis*		*R. hominis*
		*R. intestinalis*	*R. intestinalis*	*R. intestinalis*		
		*R. inulinivorans*		*R. inulinivorans*	*R. inulinivorans*	*R. inulinivorans*
				*Roseburia* sp. CAG:100		
	*Ruminococcus*	*R. gnavus*		*R. gnavus*	*R. gnavus*	*R. gnavus*
		*R. lactaris*				
		*R. obeum*				
		*R. torques*				
				*Ruminococcus* sp. CAG:177		
	*Streptococcus*					*S. agalactiae*
					*Streptococcus LJ-22*	
	*Subdoligranulum*			*Subdoligranulum* sp. 4_3_54A2FAA		
					*S. variabile*	
	*unclassified*			*Firmicutes bacterium* CAG:341; CAG:95; CAG:475		*Firmicutes bacterium* CAG:95
				*Lachnospiraceae bacterium* TF01-11; 7_1_58FAA; 9_1_43BFAA		

*gmGUS that identified to more than one species was not listed in this table.

Generally, β-glucuronidase (GUS) is a kind of glycosyl hydrolase that can specifically catalyze the hydrolysis of *O*- or *S*-glycosidic moieties and liberate the aglycones from glycosides (Awolade et al., 2020). In the GI tract, the mammalian uridine 5′-diphospho-glucuronosyltransferase [UDP-glucuronosyltransferase (UGT)] in epithelium or liver links single glucuronic acid (GlcUA) sugars to a variety of endo- and xeno-biotics. The formed glucuronide metabolites can then be transferred into the GI tract and deconjugated by gmGUS in the periplasmic space or into microbial cells. This saccharification process performed by gmGUS is not only a carbon source for maintaining gut microbiota (GM) growth but also an essential pathway for chemical biotransformation. Both exogenous and endogenous substrates with glycosidic bonds can be catalyzed into active or deactivate metabolites by gmGUS enzymatic activity (Roberts et al., 2002; Wallace et al., 2010; Chamseddine et al., 2019). Estrogen, as a very common endogenous aglycone, is metabolized into glucuronide in the liver and deconjugated in the GI tract by gmGUS (Ervin et al., 2019b; Parida and Sharma, 2019; Schiffer et al., 2019). Since estrogen has critical physiological roles in human and the overexposure of estrogen has long been considered as a determinant for sex hormone-responsive diseases such as breast cancer (BCa) (Samavat and Kurzer, 2015), this reactivation process performed by gmGUS is currently hypothesized as an important mediator for microbiota–host interaction and is also a potential link between GM and BCa (Plottel and Blaser, 2011).

Hitherto, although the biology of gmGUS has been studied and the evidence of GM (or GM metabolites)-related malignancy is accumulating, there are little published data on the interaction between gmGUS and BCa. Focusing on the gmGUS–estrogen metabolism–BCa axis, this review aims to incorporate the pieces of intricated clues, identify the remaining research gaps, and provide some recommendations for the prospective research.

## A Bidirectional Regulatory System May Exist Between gmGUS and Estrogen

β-glucuronidase was initially demonstrated by Fishman and Fishman (1944) to participate in estrogen metabolism. It was of great attention by scientists for its elevated activities observed in the malignant neoplasms of the breast, ovary, and GI tract (Fishman, 1947; Fishman and Anlyan, 1947a,b). Later, the gmGUS and its reactivating activity of estrogens have been focused on based on plausible hypotheses related to GM and BCa (Hill et al., 1971; Gorbach, 1984).

Estrogen is regarded as a major determinant of BCa and can participate in the whole process of BCa development through both estrogen receptor (ER)-dependent and -independent pathways (Sommer and Fuqua, 2001; McDonnell and Norris, 2002; Ye et al., 2008; Saha Roy and Vadlamudi, 2012), including increasing cancer cell proliferation (Tian et al., 2018; Kumar et al., 2019), stimulating angiogenesis (Gupta et al., 2007), promoting metastasis (Jiang et al., 2018), and inducing chemoresistance (Zatelli et al., 2009; Zeng et al., 2016). Furthermore, the study has shown that exogenous estrogen intervention increases the risk of BCa, whereas stopping hormone replacement therapy could significantly decrease the BCa risk (Sun et al., 2017). Epidemiological studies have also indicated that most types of BCa start as estrogen-dependent and express ER regardless of their modular subtypes. Postmenopausal women seem to be particularly sensitive to estrogen levels, and their BCa risk is directly and significantly associated with the high level of exposure to estrogens (Hankinson et al., 1998; Clemons and Goss, 2001; Key et al., 2002). For instance, Goedert et al. (2015) have detected twice higher estrogen concentrations in biopsy-proven postmenopausal BCa patients than normal-mammography women. For the crucial role and closeness with BCa, estrogen has been considered as the most potent single predictor for BCa identification (Dai et al., 2016) and has been adopted as a main molecular marker for the treatment and decision-making in patients with early BCa (Carey et al., 2006; Bauer et al., 2007; Godone et al., 2018).

**FIGURE 1:**
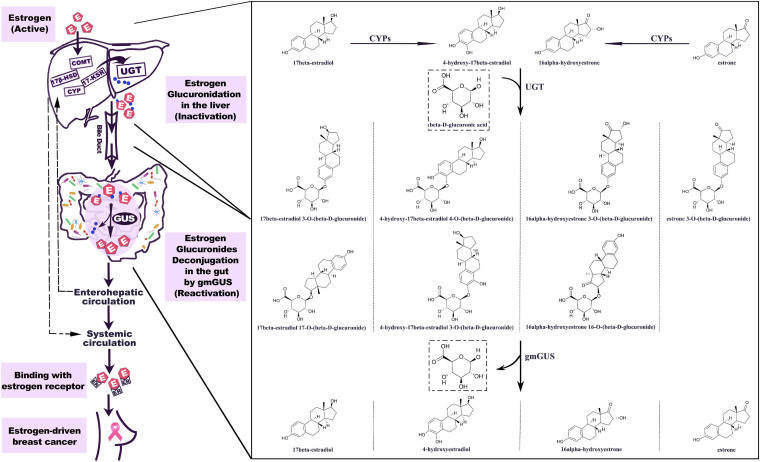
Estrogen metabolism is mediated by GUS. The hepatic metabolism of estrogen is catalyzed by a series of enzymes. Parent estrogens and the phase I metabolites can be conjugated with glucuronic acid by the catalyzation of UGT. The estrogen glucuronides are biologically inactive, but by bile excretion, they enter the gastrointestinal tract where gmGUS liberates estrogens from conjugates. The reactivated estrogens are reabsorbed into the body through the enterohepatic circulation. 7β-HSD, 17β-hydroxysteroid dehydrogenase; COMT, catechol-O-methyltransferase; CYP, cytochrome P-450 enzyme; 17-KSR, 17-ketosteroid reductase; UGT, uridine 5′-diphospho-glucuronosyltransferase; gmGUS, gut microbial β-glucuronidase; IR, insulin receptor.

**FIGURE 2:**
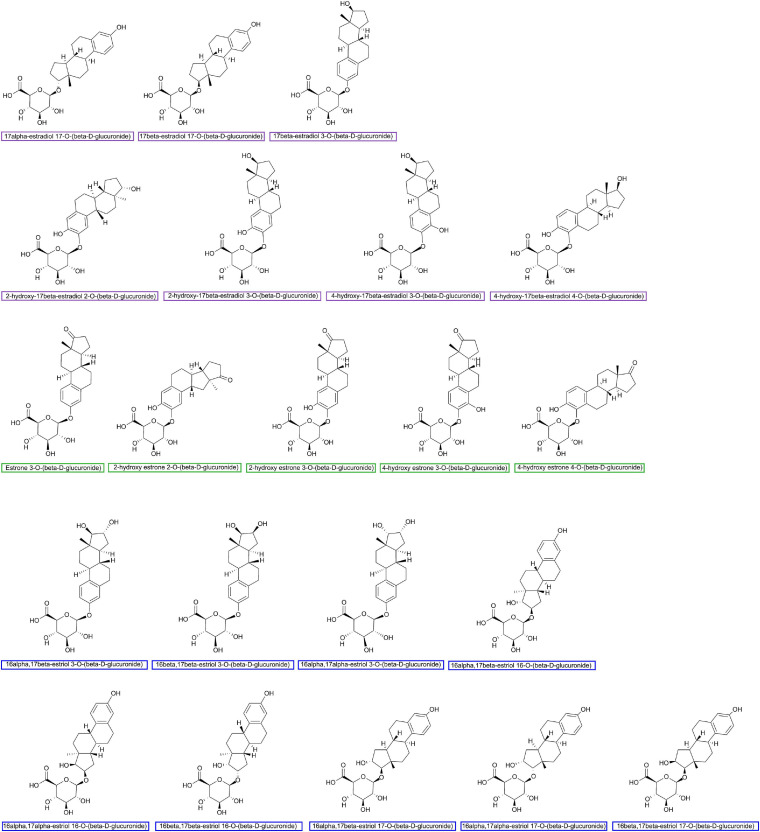
Major estrogen glucuronides finished by using ChemDraw Professional 16.0.

The important role of hepato-biliary-enteric circulation on estrogen metabolism has long been well-recognized (Emery and Joyce, 1946). Estrogens originate from C27 cholesterol and are synthesized mainly under the catalyzation of NADPH-dependent cytochrome P450 (CYP) and hydroxysteroid dehydrogenases (HSD) (Samavat and Kurzer, 2015). For premenopausal women, estradiol is the predominant form of circulating estrogen secreted by the ovaries, while for postmenopausal women, estrone is the major estrogen, synthesized in peripheral tissues such as muscle and adipose tissues (Travis and Key, 2003). The metabolism of estrogen occurs predominantly in the liver, where parent estrogens can be irreversibly hydroxylated by CYPs and then further methylated to methoxyestrogens by catechol-O-methyltransferase (COMT). Based on the different positions of hydroxylation (C2, C4, and C16), the phase I metabolites of estrogens usually perform various degrees of hormonal potency. Substantial data have proven that 2-hydroxy and 2-methoxy metabolites are weakly estrogenic and have anti-carcinoma efficiency, while 4-hydroxyestradiol and 16α-hydroxyestrone show carcinogenic potential (Clemons and Goss, 2001; Samavat and Kurzer, 2015). As shown in Figure 1, both parent estrogens and related phase I metabolites can be conjugated with glucuronic acid in 2-, 3-, 4-, 16α-, and 17-positions (Figure 2) by the catalyzation of UGT in the liver or GI epithelium (Schiffer et al., 2019). A former study showed that for young women, a large proportion of orally administrated estradiol could be converted into estradiol glucuronide, which was measured in both the blood and urine; however, the quantities observed were twice as much as those *via* i.v. administration. This result indicated that the human digestive system was an important place for estrogen glucuronidation (Longcope et al., 1985). Generally, the glucuronidated estrogens are more polar and hydrophilic and thus can easily dissolve in the blood and finally excreted through urine, but studies have found that a considerable amount of estrogen metabolites prefer entering into the GI tract through the bile excretion for further metabolism (Sandberg and Slaunwhite, 1957; Adlercreutz and Martin, 1980). In this section, gmGUS can reverse the glucuronidation process by cleaving the glucuronic moiety from estrogens, thus increasing the chance of the liberated, biologically activated estrogens to be reabsorbed through the mucosa and re-enter the circulation through the portal vein (Flores et al., 2012). In this way, gmGUS asserts its role in estrogen metabolism by modulating the enterohepatic circulation and the reabsorption process of free estrogens. With the increasing interest in GM and its role in the mammalian, in 2011, “estrobolome” was proposed to aggregate the enteric bacterial genes whose products are capable of reactivating estrogens (Plottel and Blaser, 2011). Estrobolome encoded microbe is believed to be an important mediator for estrogen metabolism by virtue of their reactivation abilities, which is mainly performed by gmGUS. The regulation mechanism of the estrobolome, especially its enzyme gmGUS on estrogen metabolism, is currently the major avenue for current GM–BCa axis research. Although as an emerging research field, rare information is found at present, and some clues indicated that there is a close connection between gmGUS and estrogen.

Similar to estrogen, gmGUS activity also seems to be related to age. Available data indicated that gmGUS activity changed with age, but there were species and sex differences. Generally, for both humans and murine, the overall gmGUS activity increased with age (Goldin et al., 1978; Mykkänen et al., 1997; Grönlund et al., 1999; Langille et al., 2014; Nowak et al., 2014), but whether this tendency is consistent among microbes is still unclear. For example, feces from 80-year-olds had higher *Lactobacillus* GUS activity but lower *Enterococcus* GUS than that from children (Mroczyńska and Libudzisz, 2010). Also, a recent study found that the change of gmGUS enzyme activity with age was in a sex-dependent manner for C57BL/6 mice. A significant decrease in enzymatic activity was associated with the increasing age in females, while no obvious connection was observed in males (Walsh et al., 2020). As the serum estradiol and estrone levels of female C57BL/6 decreased sharply with age (Nilsson et al., 2015), this intriguing finding, though preliminary, suggests that the changes in gmGUS activity may be related to the estrogen levels in the body.

Some findings have shown that estrogen intervention influenced gmGUS activity. Estrogen replacement therapy (ERT) is very efficient in decreasing the incidence of low-estrogen-caused symptoms for menopause women, but the positive connection found between the usage of ERT and BCa incidence limits its application. It is widely believed that ERT is contraindicated to women who have a high risk of BCa or have been diagnosed with BCa (Natrajan and Gambrell, 2002; De et al., 2010; Byrne et al., 2017; Lobo, 2017). Considering the important role of gmGUS in estrogen metabolism, a study investigated the impact of ERT on the gut microbiome and GUS activities. The result showed that 6 weeks of ERT intervention did not affect the overall microbiome characters on both cecal and fecal contents. However, meanwhile, the gmGUS activity test demonstrated that long-term use of conjugated estrogen/bazedoxifene significantly decreased gmGUS activity (Chen et al., 2018). Further analysis found that the decreased gmGUS activity was notably correlated with the reduced abundance of families Lactobacillaceae and Streptococcaceae, and the increased abundance of Ruminococcaceae, which are all Firmicutes with proven gmGUS activities (Kwa et al., 2016; Pollet et al., 2017). It is noteworthy that the *S. agalactiae* from Streptococcaceae, *L. rhamnosus* from Lactobacillaceae, *F. prausnitzii*, and *R. gnavus* from Ruminococcaceae have been proven to have estrogen-reactivating activities (Ervin et al., 2019b). These findings indicated the important role of gmGUS activity in the metabolism of both endogenous and exogenous estrogens, and also suggested that the disrupted gmGUS activity may be involved in ERT-induced BCa. Regarding the downregulating effect of estrogen on gmGUS discovered in this study, a recent study also found that the free form of estradiol had an inhibitory effect on *Escherichia coli* GUS activity in catalyzing the glucuronide hydrolyzation (Xiao et al., 2020). One plausible explanation for these results is that there is a bidirectional regulatory system between gmGUS and estrogen to maintain estrogen homeostasis in the body. To determine this interaction, considerable work still needs to be done.

## gmGUS Has Structure and Species Preference for Estrogen Reactivation

**FIGURE 3:**
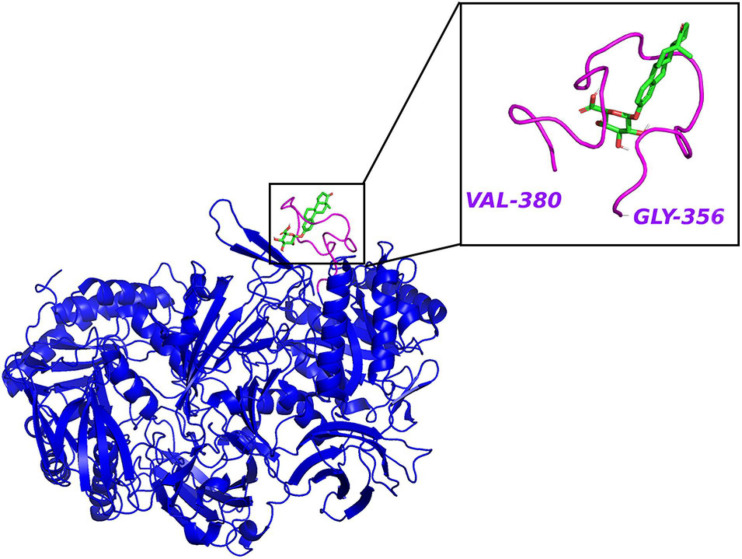
The crystal structure of *E. coli* β-glucuronidase (PDB ID: 3LPF) with estrone-3-glucuronide (PubChem CID 115255) docked in AutoDock Vina (Trott and Olson, 2010) with affinity –7.2 kcal/mol. Hydrogen atoms were added to the enzyme structure. The default docking protocol was applied and the possible poses were saved. The view of the docking results and analysis of their surface with graphical representations were done using PyMOL 2.4 (Schrodinger, 2015).

The clues from the phylogenetic distribution analysis showed that the gmGUS function was regulated by two different genes *gus* and BG, and both of them were well represented in Firmicutes while only BG was found in Bacteroidetes. The functional test showed that *gus* was the primary gene response to GUS enzyme activities, and strains carrying only the BG gene showed low activity (McIntosh et al., 2012). Activity-based probes (ABPs) have been applied to identify gmGUS from the complex fecal lysate. By synthesizing a GUS-based fluorescent probe GlcA-ABP-Atto and combining it with flow cytometry, a study identified 13 operational taxonomic units from mouse intestinal content with gmGUS activity, and they were all Firmicutes (Whidbey et al., 2019). However, the methods applied in this study may miss the community that prefers to secrete GUS to the extracellular medium rather than keeping it inside the cell. Similarly, using a biotin-ABP, Jariwala et al. (2020) revealed that the predominant gmGUS-producing species from human fecal samples are Firmicutes. Besides, recent studies have identified 279 and 444 distinct gmGUS proteins from human and mouse gut microbiome accordingly and divided them into six structural classes based on different active sites: loop 1, mini-loop 1, loop 2, mini-loop 2, mini-loop 1,2, and no loop. Intriguingly, the phyla showed their structure preference; for example, the gmGUS from Firmicutes are mainly loop 1, mini-loop 1, and no-loop types while no Bacteroidetes GUS are defined in the loop 1 category (Pollet et al., 2017; Creekmore et al., 2019). FMN binding is another type of gmGUS that showed the unique capability of small-molecule glucuronide cleavage (Ervin et al., 2019b; Pellock et al., 2019). This structure diversity among gmGUS strongly indicates that the substrate preference may exist among species and/or structure-based microbial taxonomy. Pollet et al. (2017) found that different from the mL1, L2, mL2, and NL enzymes, which were able to process polysaccharides with glucuronic acid, the L1, mL1, and L2 enzymes with longer loops processed a small glucuronide substrate more effectively. Consistently, Ervin et al. (2019b) tested 35 gmGUS activity, within which 17 of them showed the ability to reactivate estrone-3-glucuronide and/or estradiol-17-glucuronide to estrone and estradiol, respectively. Furthermore, the crystal structure analysis result showed that estrogen glucuronides belong to small-molecule glucuronides, and more likely to be processed by GUS enzymes with longer loops adjacent to the active site, such as loop 1 (Figure 3), mini-loop 1, and FMN-binding types. As Table 1 shows, after classifying the gmGUS tested in this study, we found that 18 of the 35 gmGUS are Firmicutes and 11 of them showed estrogen reactivation capacity by cleaving the glucuronic acid from both estrone and estradiol glucuronides, and *L. rhamnosus* GUS also showed a moderate deconjugating ability to estrone-3-glucuronide. However, for the 13 gmGUS from Bacteroidetes, only *B. uniformis* GUS1 (BuGUS1) and *B. fragilis* GUS showed weak activities. These consistent results indicated that Firmicutes gmGUS containing loop 1, mini-loop 1, and FMN structures was the primary source of estrogen-responsive gmGUS.

## Site Prediction for GUS Performing Estrogen Reactivation

The location and regional abundance of gmGUS are mainly determined by the microbial distribution. The spatial and temporal non-uniformity of GM combined with the species and structural diversity of gmGUS indicate that the function of gmGUS is more likely to have substrate specificity and regional character. In this setting, considering that small-molecule glucuronides, such as endogenous sex hormone and bilirubin glucuronides, are currently the main focused Phase II metabolites, which are usually catabolized in the small intestine and accordingly reabsorbed *via* the intestinal epithelia to realize their enterohepatic circulation (Pellock and Redinbo, 2017; Little et al., 2018; Martinez-Guryn et al., 2019), it is rational to propose that the small intestine especially the jejunum and ileum are probably the main places for gmGUS to perform enzymatic activities, especially to small-molecule glucuronides such as estrogen.

Although there is currently no conclusive evidence to support this hypothesis, some studies on the regional distribution of GM can provide support. Generally, Bacteroidetes is the dominant phyla of the large intestine for its degradation ability of complex carbohydrates by producing polysaccharide utilization loci-organized gmGUS and other carbohydrate-active enzymes, while Firmicutes and Proteobacteria mainly locate in the small intestine and compete with the host for nutrient by virtue of gmGUS activity (Dabek et al., 2008; Zoetendal et al., 2012; Kwa et al., 2016; Grondin et al., 2017; Pellock and Redinbo, 2017). A study revealed that Bacteroidetes bacteria *B. thetaiotaomicron* has a “pedal bin” substrate transport system by starch utilization system protein-formed complexes SusCD to import the outer membrane high-molecular-weight glycans into the periplasmic space (Glenwright et al., 2017). Also, a study found that some Proteobacteria microbes such as *E. coli* encode transcriptional repressor GusR orthologs, thus controlling the GUS operons and promoting bacterial glucuronide recognition capacity in the small intestine (Little et al., 2018). Although a similar regulation system of firmicutes has not yet been discovered, the GM distribution strengthens the hypothesis that estrogen glucuronides are mainly reactivated by Firmicutes gmGUS in the small intestine.

The gmGUS-specific probe seems to be a powerful tool for *in situ* imaging of gmGUS in the GI tract, but currently, only a few studies have focused on it and the results were rather uncertain. A fluorescein Di-β-D-Glucuronide probe showed that the large intestine was the major area of gmGUS in the mouse intestine (Chen et al., 2017). However, the NIR fluorescent probe designed by Feng et al. (2018) detected the highest fluorescence intensities in the ileum and final jejunum of the mouse. The subject-related factors (such as sex, age, and diet regimen) and the probe efficiency are partly responsible for the discrepancy. For *ex vivo* analysis, a previous study estimated that GUS activities in proximal and distal regions of the human small intestine were 0.02 and 0.9 μmol of substrate degraded/h/g content, respectively (Hawksworth et al., 1971). Sakamoto et al. (2002) compared the GUS activities of different intestinal contents of rats. The result showed that cecum content was responsible for about 70% of total gmGUS enzyme activity, and the remaining percentage was mainly performed by colon contents. Moreover, the author tested the metabolism of xenoestrogen bisphenol A (BPA) in the digestive tract of rats and found that BPA glucuronide was rapidly synthesized after orally administered (15 min), but its distribution was only limited to the small intestine. A large amount of free BPA was later detected in the cecum while the contents dropped rapidly in the colon and feces. The result further indicated that the distal small intestine and cecum is the major place for gmGUS catalyzation and hydrolyzate reabsorption.

Besides, studies found that pH is an important factor for gmGUS activity (Eichenbaum et al., 2012; Pollet et al., 2017; Feng et al., 2018). With the same substrate *p*-nitrophenol-β-D-glucuronide (*p*NPG), the optimal pH for *E. coli* GUS, *Bacteroides fragilis* GUS, and *Faecalibacterium prausnitzii* GUS were 7.4, 5.0, and 6.0, respectively (Biernat et al., 2019; Creekmore et al., 2019). For this reason, the optimal pH may be a meaningful indicator of the distribution and function of gmGUS. Ervin et al. (2019b) selected several gmGUSs with processing estrogen glucuronides capacity and found that their optimal pH for catalyzing *p*NPG hydrolysis was mainly between 5.5 and 6.5, which was consistent with the pH of the small intestine (Evans et al., 1988; McConnell et al., 2008; Zoetendal et al., 2012; Koziolek et al., 2015; Amara et al., 2019). However, a note of caution is due here since the optimal pH for gmGUS may vary with the substrates. Even for the same type of substrate (both are small-molecule glucuronide substrates), gmGUS could exhibit significantly different activities under the same pH (Biernat et al., 2019), so the optimal pH for the estrogen-deconjugating capacity of gmGUS needs to be specifically determined before being applied for gmGUS position prediction.

The findings reported here suggest the hypothesis that estrogens are mainly reactivated by Firmicutes gmGUS containing loop 1, mini-loop 1, and FMN structures in the small intestine. Further research is required to establish the full atlas of gmGUS in the GI tract and the precise sites of the deconjugation of estrogen glucuronide.

## The High-Fat Diet–gmGUS–BCa Axis

Previous research has proposed the existence of the high-fat diet (HFD)–GM–BCa axis. Shapira et al. (2013) estimated that the increased estrogen bioactivity caused by HFD-induced GM dysbiosis may be responsible for a 20% higher risk of BCa. Some studies also posed the hypothesis that HFD promotes BCa by upregulating the gmGUS activity and consequently resulting in increased circulating estrogen levels (Yang et al., 2017; Kang et al., 2018). However, most of the evidence for this plausible relationship is fragmentary and indirect, so it needs to be gathered and interpreted with caution.

A HFD has long been discussed as a potent factor in BCa. Though the complex mechanisms are still not fully understood (Jiralerspong and Goodwin, 2016), the endogenous estrogen metabolism is arguably at the forefront (Arnold et al., 2015; Keum et al., 2015; Engin, 2017), especially for women in post-menopause, when the circulating estrogen is largely produced by extra-glandular tissues such as adipose tissue (Travis and Key, 2003). A cross-sectional study found that body mass index was positively correlated with circulating free estrogen levels, but negatively correlated with conjugated estrogens in postmenopausal women (Fuhrman et al., 2014). Another clinical study suggested that high total and saturated fat were positively associated with a greater risk of ER-positive BCa (Sieri et al., 2014). Besides, low-density lipoprotein, which can be adversely elevated by a HFD, is the main source of estrogen precursor C27 cholesterol (Samavat and Kurzer, 2015). The study indicated that the administration of lipophilic statins (clinical cholesterol-lowering drugs) effectively promoted the treatment of ER-positive BCa in postmenopausal women (Desai et al., 2015).

The dominating role of diet to GM suggested that diet was the most critical and also controllable environmental factor to gmGUS activity (Grzelak-Blaszczyk et al., 2018; Konieczka et al., 2020). Although the mechanism is still elusive, a considerable number of studies have reported that HFDs could promote gmGUS activity (Reddy et al., 1977, 1980; Grzelak-Blaszczyk et al., 2018, 2020). Rats feeding on high corn oil or lard oil diet had higher gmGUS activities in both cecum and colon contents than rats with a normal diet (Reddy et al., 1977; Wu and Chen, 2011). Rabbits with 4 weeks of HFD showed significantly increased cecal GUS activity (Jurgoński et al., 2014). Diet-induced obese mice also showed about twice higher fecal GUS activity than lean controls (Mallick et al., 2015). Healthy adults had increased fecal GUS activity after consuming a high-fat dietary regimen for 4 weeks (Reddy et al., 1980). Besides, the study also suggested that the source of fat influenced the outcome. Fat from meat seems to be more favorable to the increase of gmGUS activity than that from dairy products (Reddy et al., 1978). Notably, some findings highlighted the effect of HFD on estrogen-related gmGUS activity. Recently, there is an abundance of studies focusing on GM perturbation or dysbiosis that happened during HFD intervention. Even though the results were not always consistent, the increase in *Firmicutes* and decrease in *Bacteroidetes* or increased *Firmicutes*-to-*Bacteroidetes* ratio are considered as an important sign of obesity (Sekirov et al., 2010; Daniel et al., 2014; Gilbert et al., 2016; John and Mullin, 2016; Luu et al., 2017). Correspondingly, a study found that mice fed on a HFD showed a larger proportion of loop 1 and no-loop gmGUS than that of low-fat-fed mice, and both types of GUS are predominantly from Firmicutes (Creekmore et al., 2019). As previously stated, Firmicutes gmGUSs with a loop 1 structure were outstanding in estrogen reactivation (Ervin et al., 2019b). Together, these studies provide valuable insights into the interactions between HFD, gmGUS, and BCa. In future investigations, definitive evidence to clarify the causality and accuracy of this axis should be a focus.

## Strategy to Uncover the Unknown

Even though the evidence is rapidly accumulating about the role of GM in the occurrence and development of diseases, a large proportion of these data are elusive and inconsistent, especially when considering the ability to clarify whether the altered GM or gmGUS is the consequence of the disease process or is somehow involved in its pathogenesis. In this case, though it seems plausible to hypothesize that GM takes part in BCa development by the estrogen reactivating activity of gmGUS, the impact is still ambiguous due to the exquisite estrogen homeostasis system and the complicated relationship between estrogen metabolism and BCa. Until now, data about the connection between gmGUS and BCa development is very limited and lacks relevance, but hopefully, a lot of studies are focusing on this field. To navigate the exploring process and fuse the fragments into a cohesive whole, some strategies are proposed here.

### Clinical Evidence

To make the hypothesis more rational and stable, clinical studies about the gmGUS activity in estrogen dysbiosis-related diseases, such as hyperestrogenism-related breast/endometrial cancer and hypoestrogenism-related osteoporosis, need to be conducted. For example, a study tested the connection of gmGUS activity with non-ovarian estrogen metabolism in healthy postmenopausal women and found that fecal gmGUS activity was positively connected with urine estrone level but negatively related to both free and conjugated estrogen levels in feces. However, no significant connection was observed between gmGUS and urine estradiol/total estrogen/total metabolites levels (Flores et al., 2012). The small sample size and limited reference data make the results difficult to explain, but as the pioneer in this area, this study is still noteworthy for reminding us that there is a huge gap of data in the clinical research for verifying the relationship between gmGUS, estrogen metabolism, and BCa. In this setting, well-designed longitudinal and cross-sectional studies are expressly required to provide clinical evidence to support the hypothesis and valuable insights for further fundamental research.

### Next-Generation Gut Microbiota Gene Sequencing Technology

The change of GUS activity is not only the induction of enzyme synthesis but also the reflection of GM perturbation. As the GM community is being rapidly mapped out and confirmed, the construction of GM may also become a power indicator to identify the activity of gmGUS. In this case, the abundance and diversity of the estrobolome need to be closely monitored during tumorigenesis and tumor growth. The association between the altered gut microbiome and BCa was reported as early as 1990 when a study compared the GM of 7 healthy women and 11 women with BCa (Minelli et al., 1990). In recent years, 16S rRNA gene sequencing and metagenome sequencing have been widely applied in microbial-related research (Gopalakrishnan et al., 2018; Zhu et al., 2018). For GM, these technologies not only provide the overall image of the GM diversity and abundance but also sensitively screen out the target taxa with specific properties. For example, by using these technologies, a study tested the connection between urinary estrogens and fecal microbiome of 60 postmenopausal women. It found that the ratio of metabolites to parent estrogen was positively correlated with microbial phylogenetic diversity (Fuhrman et al., 2014), but the paper lacks further analysis of the function of the specific taxa and their connection with BCa. In another study, by using 16S rRNA sequencing, the author found that BCa patients with BMI over 25 kg/m^2^ had lower microbiota abundance in stools than that of normal-weight subjects, and the abundance of GM species with gmGUS activity (*C. coccoides* cluster, *C. leptum* cluster, and *F. prausnitzii*) was significantly higher in more severe BCa clinical-stage group in which over 90% of patients were hormone receptor-positive (Luu et al., 2017). However, the author did not quantify the gmGUS activity specifically, which resulted in the results lacking credibility. These studies suggest that advanced sequencing technologies can not only provide substantial information about the taxonomic diversity of GM but also give an indication of the GM metabolic potential under different situations. For gmGUS research, these technologies can be fully utilized by combining them with further mechanism study.

### Pharmacological Intervention by the Specific Enzyme Inhibitor

Pharmacologically up- or downregulating enzyme activity is a useful tool to investigate gmGUS functions. Mammalian GUS inhibitor, as a hopeful anti-tumor drug, was proposed as early as 1949 (Karunairatnam and Levvy, 1949; Boyland et al., 1957). In recent years, the severe and high incidence of drug-induced GI toxicity such as chemotherapy drug irinotecan (and its toxic metabolite SN-38) caused delayed diarrhea (Bhatt et al., 2020) and has been associated with the deconjugation activity of gmGUS. For this reason, the potential gmGUS inhibitors were first proposed in the 2000s (Lötsch et al., 2002) and accumulated rapidly after Wallace et al. (2010) determined and refined the *E. coli* β-glucuronidase structure. Table 2 summarizes some clinical drugs and natural products that have been proposed since 2019 with anti-gmGUS activity, but even the number of inhibitors continues to grow, and there is still no validated intestinal GUS inhibitor in clinical use (Chamseddine et al., 2019), so developing potent and sensitive inhibitors is currently a major area of interest within the gmGUS field.

**TABLE 2:** Summary of potential gmGUS inhibitors reported since 2019.

**Inhibitor**	**Type**	**Evidence**	**References**
Chang-wei-qing	Chinese herbal formula	*Ex vivo* fecal GUS inhibition assay	Wan et al., 2019
Lycopene	Dietary bioactive compound	*Ex vivo* fecal, cecal, and colonic content GUS inhibition assay	Valadez-Bustos et al., 2019
*Bifidobacterium longum*	Probiotic		
Raloxifene and analogs	Drug; Synthetic compounds	High-throughput screening assay; *ex vivo* cecal GUS inhibition assay	Ervin et al., 2019a
Vancomycin	Drug	*Ex vivo* intestinal content GUS inhibition assay; *ex vivo* gmGUS-related GM detection	Whidbey et al., 2019
		*Ex vivo* fecal GUS inhibition assay; *ex vivo* imaging of intestinal GUS activity	Taylor et al., 2019
Pyrazolo[4,3-c]quinoline derivative	Synthetic compounds	Cell-free/Cell-based bacteria GUS inhibition assay; *in vivo* intestinal GUS inhibition assay	Cheng et al., 2019
*Sorbus* leaf extract	Natural products	Cell-free *E. coli* GUS inhibition assay	Kavak and Akdeniz, 2019
Demethoxydaibucarboline A, quercetin, methyl-neolitacumone A, and epicatechin	Natural products from *Neolitsea acuminatissima*	Cell-free human GUS and *E. coli* GUS inhibition assay	Lin et al., 2020
Cinnamic acid derivatives	Natural products	Cell-free *E. coli* GUS inhibition assay; molecular docking	Li et al., 2020
Flavonoids	Natural products from *Primula boveana* leaf	HPTLC-UV/Vis/FLD-*E. coli* GUS inhibition assay system	Mahran et al., 2020
Silychristin and silybin	Natural products from *Silybum marianum*		
Thiazolidin-2-cyanamide derivatives	Synthetic compounds	Cell-free *E. coli* GUS inhibition assay; molecular docking	Zhou et al., 2020
(7S,8S,7′R,8′R)-isoamericanol B and americanol B; moricitrins A and B	Natural sesquineolignans and natural dineolignans from noni fruits	Cell-free *E. coli* GUS, α-amylase, α-glucosidase, and pancreatic lipase enzyme inhibition assay	Yang et al., 2020
Demethylbellidifolin	Natural flavonoids	Cell-free *E. coli* GUS inhibition assay; molecular docking and molecular dynamics simulations	Sun et al., 2020
Gentisin			
70% nixtamalized corn (*Zea mays* L.)/30% cooked common bean (*Phaseolus vulgaris* L.) snack	Snack	*Ex vivo* fecal GUS inhibition assay	Luzardo-Ocampo et al., 2020
Jiawei Xianglian Decoction	Traditional Chinese Medicine	*Ex vivo* fecal GUS inhibition assay	Lu et al., 2020
Quercetin diglucosides preparation	Natural products from yellow onion	*Ex vivo* extracellular and intracellular cecal GUS inhibition assay; *ex vivo* Fecal GUS inhibition assay	Grzelak-Blaszczyk et al., 2020
Iminocyclitols and analogs	Synthetic compounds	*R. gnavus* GUS, *B. fragilis* GUS, *C. perfringens* GUS, *E. coli* GUS, and bovine liver GUS inhibition assay	Dashnyam et al., 2020
N-desmethylclozapine, Aspartame, and Gemifloxacin	Drug	Cell-free/Cell-based *E. coli*/bovine GUS inhibition assay; molecular docking and molecular dynamics simulations	Chen et al., 2020
VSL#3^®^	Probiotic	*Ex vivo* fecal GUS inhibition assay	dos Santos Cruz et al., 2020
VSL#3^®^ and yacon diet	Synbiotic		
Flavonoids	Natural products from Mulberry bark	Cell-free *E. coli* GUS inhibition assay; molecular docking	Bai et al., 2021
Hypochlorite and peracetic acid	Chemical compounds	Cell-free *H. pomatia* GUS inhibition assay	Zhong et al., 2021
Apple pectins	Enzymatically isolated from dried apple pomace	Cell-free endogenous *E. coli* GUS inhibition assay	Palko-Łabuz et al., 2021
PectaSol-C	Commercially available modified Citrus pectin		
Amentoflavone	Natural product from *Selaginella tamariscina*	Cell-free/Cell-based *E. coli* GUS inhibition assay; molecular docking and molecular dynamics simulations	Tian et al., 2021
Uronic isofagomine derivatives	Synthetic compounds	Cell-free human GUS, *E. coli* GUS, *B. dentium* GUS, *C. perfringens* GUS, and *L. gasseri* GUS inhibition assay; cell-based *E. coli* GUS inhibition assay; *in vivo* intestinal GUS inhibition assay	Lin et al., 2021
Cricket powder	Natural food additive	*Ex vivo* intestinal contents GUS inhibition assay	Kowalczewski et al., 2021
Isoprenylated chromane derivatives	Natural products from coculture of *Pestalotiopsis* sp. and *Penicillium bialowiezense*	Cell-free *bacterial* GUS inhibition assay	Li et al., 2021
Melittin and cecropin A	Natural antimicrobial peptides from insect	*Ex vivo* total, extracellular, and intracellular fecal GUS inhibition assay	Juśkiewicz et al., 2021
Lactoferrin	Natural antimicrobial protein		

The high sequence similarity between gmGUS and orthologous mammalian GUS and the biological diversity of GM and its metabolites in the GI tract may be partly responsible for the delay. Mammalian GUS is an essential lysosomal enzyme. A deficiency or reduced activity of human GUS can result in lethal lysosomal storage disease mucopolysaccharidosis type VII (Sly Syndrome) (Paigen, 1989). GUS enzyme from *E. coli* shares overall 50% sequence with the human lysosomal enzyme (Jain et al., 1996) and 45% sequence identity with human GUS (Wallace et al., 2010). This similarity may lead to the gmGUS inhibitors with low specificity easily targeting the mammalian GUS, thereby causing severe side effects. In this case, the “bacterial loop,” which is a bacteria-specific structure missing in the orthologous mammalian GUS, has been used as a useful target for developing bacterial GUS-specific inhibitors (Wallace et al., 2010; Kong et al., 2014; Jariwala et al., 2020). For example, by using bovine liver β-glucuronidase as a negative control, four potent gmGUS inhibitors were screened and showed potent efficacy by inhibiting the β-glucuronidase enzyme activity both *in vitro* and in living bacterial cells without affecting bacterial cell growth or survival. Furthermore, the high specificity of these inhibitors relies on the “bacterial loop” of gmGUS. Thus, no effect of the inhibitors was observed on mammalian β-glucuronidase activity and also mammalian epithelial cells. *In vivo* study further showed that one of the inhibitors could effectively eliminate the delayed diarrhea and intestinal damage caused by CPT-11 (irinotecan) administration. This study highlighted the significance and possibility of gmGUS-targeted inhibitors in clinical application and also indicated that evaluating the effect of gmGUS inhibitors on mammalian GUS is necessary to develop inhibitors with potent efficacy and high specificity (Wallace et al., 2010). Besides, it should be noted that gmGUS is not the only glycoside hydrolase in the GI tract; β-glucosidases, β-galactosidases, and β-mannosidases, which share similar structures and functions with gmGUS, may be potential off-target hits for gmGUS inhibitors (Pellock et al., 2018; Jariwala et al., 2020).

Furthermore, as we discussed before, the structure of gmGUS has species difference, which indicates the limitation of most studies using single species, such as *E. coli* GUS, as the target GUS to synthesize or screen microbial gmGUS inhibitors regardless of considering the functional and structural consistency between it and the target gmGUS. Therefore, it should be noted that to develop potent and selective inhibitors for target gmGUS, the propensity of the inhibitor should be considered, and the efficiency of the inhibitor to both the entire and the target gmGUS activity should be systemically evaluated. For example, in a recent study, four bacterial GUS were applied to evaluate the potency and selectivity of inhibitors. Only one of six inhibitors has shown high selectivity for opportunistic bacterial GUSs (*E. coli* GUS and *C. perfringens* GUS) while all others showed universal inhibition to gmGUS. The various active-site loops of the gmGUS structure led to the different binding affinities between inhibitors and enzymes (Dashnyam et al., 2020). Another study also showed that gmGUS with loop 1 structure could process SN38-G more effectively than other types, based on which, the loop 1-targeted GUS inhibitor UNC10201652 was synthesized and showed great activities in blocking irinotecan-induced intestinal damage (Bhatt et al., 2020). In this regard, structure-based high-throughput screening may be a useful strategy for gmGUS inhibitor development (Wallace et al., 2010; Dashnyam et al., 2018; Feng et al., 2018; Chen et al., 2020; Jariwala et al., 2020).

### High-Sensitivity Probe for GUS Distribution Imaging and Activity Evaluation

The non-standardized fecal collection procedures and gmGUS activity assay protocol limit the reliability of the results and make the data inconsistent. The pH and location preference of gmGUS discussed earlier further suggested the deficiency of current *ex vivo* and *in vitro* tests. For these reasons, gmGUS activity-based probes are expressly needed for the real-time distribution imaging and activity evaluation of gmGUS *in vivo*. In addition to gmGUS-related GM identification, the GlcA-ABP-Atto probe designed by Whidbey et al. (2019) has also been applied as a functional approach to detect the influence of vancomycin on gmGUS activity. Jariwala et al. (2020) have successfully applied the cyclophellitol-based GUS ABPs to target gmGUS from complex systems (enzyme mixture, mouse fecal, and human fecal). After combining with LC-MS/MS and a bioinformatics pipeline, an ABPP-enabled proteomics pipeline was then developed and used to provide a more detailed profile of the gmGUS in samples. The obtained GUS abundance information was further applied for the discovery of SN-38 processing gmGUS. The author found that SN-38 reactivating *in vivo* was highly correlated with the abundance of gmGUS with a loop 1 structure, and the *E. eligens* GUS was the key regulator processing this reaction. This study highlighted the high efficiency of ABPs in identifying and quantifying the abundance and bioactivity of gmGUS. Intriguingly, gmGUS probe also showed its versatility in *in vivo* imaging and gmGUS inhibitor screening. By combining with the HPLC/MS technique, the probe-based high-throughput screening system was established and successfully identified one potential gmGUS inhibitor, (−)-epicatechin 3-gallate, from a series of herb extracts effectively (Feng et al., 2018). The high flexibility and compatibility of the existing probes indicate the considerable research potential and broad applicability of gmGUS-targeted probes. Thus, developing probes with estrogen glucuronide as specific substrates will remarkably advance our understanding of the interaction between gmGUS and BCa, though the *in vivo* safety and stability and the target specificity and sensitivity must be critically evaluated before being applied in the clinic.

## Discussion

The data for the role of gmGUS in BCa is continually updated, and increasing findings suggest the existence of the gmGUS–estrogen–BCa axis. However, up till now, studies about gmGUS activity for estrogen metabolism and BCa are still scarce, and a fair degree of inconsistency exists among them, which makes much of the evidence reported here circumstantial. For this reason, more systematic studies are needed to fuse these fragments into a cohesive whole, and readers are reminded to interpret these data with caution and judgment.

The top concern for this field is about the importance of the regulation activity shown by gmGUS to the homeostasis of estrogen in the body, and the possibility to connect this effect to BCa. More than 60 years ago, Sandberg and Slaunwhite (1957) found that, for both healthy and carcinoma patients, over 50% of i.v. injected estradiol and estrone could be excreted in the bile, and most of them were present in the conjugated fraction. Still, disappointingly, the amounts of glucuronide metabolites in bile were small. Combining with the high concentration of estrogen glucuronides detected in the urine, the authors proposed that instead of being excreted in the bile and reabsorbing in the GI tract, glucuronide estrogens were mainly generated from the reabsorbed estrogens, which have finished the deconjugated process and re-entered into the liver through enterohepatic recirculation. In this case, the role of gmGUS in the estrogen reactivation process seems negligible, and this may partly explain the undesirable result of a recent study in which the loop 1 GUS-specific inhibitor with effective *in vitro* activity to estrogen glucuronides has a limited anti-tumor effect on the estrogen-responsiveness BCa animal model (Marjon et al., 2014; Ervin et al., 2019b).

Besides, when animal models seem to be the most common and useful tool for studying the interactions between humans and their intestinal inhabitants, reliability and applicability must be considered. Several studies have uncovered the variability of gmGUS in different animals and mentioned that gmGUS activity is much higher in rat and mouse GI tract than in the human intestine (Drasar, 1974; Rowland et al., 1986; Walsh et al., 2020). Hundreds of gmGUS have been identified in both human and mouse intestine, but less than 10% of them are highly similar. The differences can be observed in both the structure category proportion and microbial functions (Creekmore et al., 2019), and coupled with the unavoidable impacts of strain, age, diet, and also the intestinal sampling position to the consistency of the results (Hawksworth et al., 1971; Son et al., 2019; Walsh et al., 2020), a multi-strategy is highly recommended when using animal models for gmGUS research.

Last but not least, special attention should also be paid to the sample preparation and substrate selection process for gmGUS enzymatic activity detection. The cellular location of gmGUS performing enzyme activity is related to its protein structure and the species of GM. Pollet et al. (2017) estimated that gmGUS with a loop 1 structure was more likely to be intracellular for the lack of N-terminal signal sequence, while loop 2, mini-loop 2, and mini-loop 1,2 gmGUS could be transferred to periplasmic space. Also, the mini-loop 1 and no-loop gmGUS secreted by Firmicutes are intracellular, but the same types of gmGUS from Bacteroidetes could be transported across the inner microbial membrane. Therefore, different sample preparation methods (lysing cells or not) may affect the consistency of results. Besides, as the enzymes have substrate selectivity, though *p*NPG is currently the most common chromogenic substrate for the *ex vivo* and *in vitro* gmGUS tests, estrogen glucuronides shown in Figure 2 are recommended as logical substrates to evaluate the estrogen reactivation activity of gmGUS (Ervin et al., 2019b).

## Conclusion

The literature review concentrates largely on the possible link between gmGUS and BCa. Focusing on the protein structure, related GM community, intestinal distribution, and enzyme function of gmGUS, a series of clues are summarized here and interpreted cautiously to provide conclusive evidence to the hypothesis that the estrogen reactivating activity of gmGUS is the way for gut microbiota to participate in BCa. However, our understanding of this field is superficial and still at its early stages. The precise mechanism of the interaction between gmGUS and estrogen metabolism remains to be elucidated, and more empirical evidence for the connection between gmGUS and BCa and their causality is required.

## Author Contributions

YS wrote the manuscript. JW revised the manuscript. JC contributed to the revision and approved the submitted version. All authors contributed to the article and approved the submitted version.

## Conflict of Interest

The authors declare that the research was conducted in the absence of any commercial or financial relationships that could be construed as a potential conflict of interest.

## Publisher’s Note

All claims expressed in this article are solely those of the authors and do not necessarily represent those of their affiliated organizations, or those of the publisher, the editors and the reviewers. Any product that may be evaluated in this article, or claim that may be made by its manufacturer, is not guaranteed or endorsed by the publisher.
